# A general but still unknown characteristic of active oxygen evolution electrocatalysts†

**DOI:** 10.1039/d2sc06832j

**Published:** 2023-03-07

**Authors:** Eleonora Romeo, Francesc Illas, Federico Calle-Vallejo

**Affiliations:** a Department of Materials Science and Chemical Physics, Institute of Theoretical and Computational Chemistry (IQTCUB), University of Barcelona C/Martí i Franquès 1 08028 Barcelona Spain francesc.illas@ub.edu; b Nano-Bio Spectroscopy Group, European Theoretical Spectroscopy Facility (ETSF), Department of Polymers and Advanced Materials: Physics, Chemistry and Technology, University of the Basque Country UPV/EHU Av. Tolosa 72 20018 San Sebastián Spain federico.calle@ehu.es; c IKERBASQUE, Basque Foundation for Science Plaza de Euskadi 5 48009 Bilbao Spain

## Abstract

The unsatisfactory electrocatalysis of the oxygen evolution reaction (OER) is a major hurdle for the sustainable production of hydrogen using water electrolyzers. Besides, most state-of-the-art catalysts are based on expensive and scant elements such as Ru and Ir. Hence, it is paramount to establish the features of active OER catalysts to make well-informed searches. Here, an affordable statistical analysis exposes a general yet unnoticed characteristic of active materials for the OER: they frequently have three out of four electrochemical steps with free energies above 1.23 eV. For such catalysts, the first three steps (abbreviated as: H_2_O → *OH, *OH → *O, *O → *OOH) are statistically prone to be over 1.23 eV, and the second step is often potential limiting. Finally, “electrochemical symmetry”, a recently introduced concept, is shown to be a simple and convenient criterion for the *in silico* design of enhanced OER catalysts, as materials with three steps over 1.23 eV tend to be highly symmetric.

## Abstract 2

A common feature of active catalysts for oxygen evolution is that three of their four electrochemical steps are over 1.23 eV. This evidences that “electrochemical symmetry” is a good criterion to design enhanced OER catalysts.

## Graphical abstract

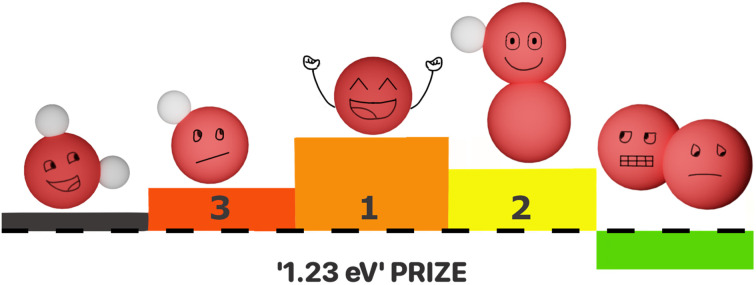

## Introduction

Our goal in this contribution is to uncover a general but still unknown energetic feature of active catalysts for the oxygen evolution reaction (OER, in acidic conditions: 2H_2_O → O_2_ + 4H^+^ + 4e^−^). This reaction displays large overpotentials, particularly in acidic media,^1–3^ and is catalyzed by materials typically based on ruthenium and iridium, which are both expensive and scarce elements on the Earth's crust.^4^ Arguably, the deficient electrocatalysis of the OER is a foremost impediment on the way toward the global, sustainable production of hydrogen using water electrolyzers.

To reach our goal, several ingredients are necessary. First of all, we need a considerable amount of data, such that statistically meaningful analyses can be made. Table S1 in the ESI† shows all the data we collected from the literature for single-atom catalysts (SACs),^5,6^ monoxides (MO),^7–9^ dioxides (MO_2_),^8^ doped and undoped TiO_2_,^10^ perovskite oxides (BaNiO_*x*_,^11^ Sr_*x*_Na_*y*_RuO_3_,^12^ LaMO_3_ and SrMO_3_ (ref. 7–9)), double perovskites (Sr_2_MIrO_6_ (ref. 13) and LSNMR^14^), and metalloporphyrins.^15^ In total, the set includes around 160 data points from different materials, the structures of which were optimized using density functional theory (DFT) calculations with functionals of the generalized gradient approximation (GGA) family.

Second, we need a means of describing the energetics of proton–electron pairs in electrochemical media. Here we adopted the computational hydrogen electrode approach,^16^ which seizes the equilibrium in solution between gas-phase hydrogen and protons and electrons, such that: 
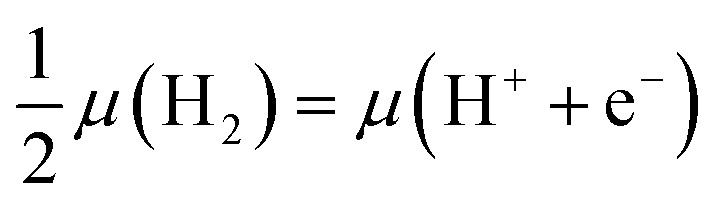
. This allows us to calculate the adsorption energies of the intermediates and the free energies of the electrochemical steps within a simple and self-consistent thermodynamic framework.

Third, we need a catalytic pathway connecting the reactants and products of the OER. In this case we opted for the most widely used pathway in the literature,^8,17^ see eqn (1)–(4).

1 H_2_O + * → *OH + H^+^ + e^−^

2 *OH → *O + H^+^ + e^−^

3 *O + H_2_O → *OOH + H^+^ + e^−^

4 *OOH → * + O_2_ + H^+^ + e^−^

where a lone asterisk denotes a free site at the catalyst surface, and an asterisk next to an intermediate implies that the species is adsorbed. We note in passing that other electrocatalytic pathways involving for examples lattice oxygen and chemical steps have been proposed in the literature^18–23^ but go beyond the scope of this work. As shown below, the reaction energies of the electrochemical steps can be expressed in terms of those of the three adsorbed intermediates, namely, *O, *OH and *OOH (Δ*G*_O_, Δ*G*_OH_, Δ*G*_OOH_).

5 Δ*G*_1_ = Δ*G*_OH_

6 Δ*G*_2_ = Δ*G*_O_ − Δ*G*_OH_

7 Δ*G*_3_ = Δ*G*_OOH_ − Δ*G*_O_

8 Δ*G*_4_ = Δ*G*_O_2__ − Δ*G*_OOH_

where Δ*G*_O_2__ is equivalent to the total energy of the reaction, which is 4.92 eV in experiments at standard conditions. This number is the product of the equilibrium potential of the reaction (*E*^0^ = 1.23 V *vs.* RHE) times the number of transferred electrons (4 e^−^). Before continuing, we note that ample discussions on the use of 4.92 eV instead of the DFT-calculated values (which tend to underestimate the experimental value) are provided in other works, where the effects of this choice on free-energy diagrams and Sabatier-type activity plots are also illustrated.^24,25^ In addition, Δ*G*_OH_, is defined with respect to water and proton–electron pairs as shown in eqn (1) and (5), while Δ*G*_O_ and Δ*G*_OOH_ are the free energies of reaction of eqn (9) and (10), respectively.

9 H_2_O + * → *O + 2H^+^ + 2e^−^

10 2H_2_O + * → *OOH + 3H^+^ + 3e^−^

Fourth, we need a metric for the OER activity. The most widespread in computational electrocatalysis is perhaps the “thermodynamic overpotential”,^8,26^ calculated on the basis of eqn (5)–(8) as:

11 *η*_OER_ = max(Δ*G*_1_, Δ*G*_2_, Δ*G*_3_, Δ*G*_4_)/e^−^ − *E*^0^

The step with the largest (most positive) free energy change is called the potential-limiting step (PLS). Moreover, it is worth noting that the concept of potential-limiting step is different from that of rate-determining step. The former is a thermodynamic construct whereas the latter is a kinetic one. Nonetheless, they are often connected in electrocatalytic pathways,^27,28^ and we will assume their correspondence in the analysis that follows.

Finally, we need some descriptors to capture the OER activity trends. For our current purposes, we will only consider three descriptors, but we stress that several other experimental and computational descriptors are available in the literature.^29–34^ The first one is probably the most widely used descriptor in the literature and corresponds to the difference between the adsorption energies of *O and *OH (*i.e.*, Δ*G*_O_ − Δ*G*_OH_,^2,8^ which corresponds to Δ*G*_2_, see eqn (2) and (6)). Furthermore, the scaling relation observed on a vast number of materials^7,8,15,26^ is such that: Δ*G*_OOH_ ≈ Δ*G*_OH_ + 3.20. However, it should ideally be Δ*G*_OOH_ = Δ*G*_OH_ + 2.46, given that *OH and *OOH are separated in the catalytic pathway by two electron transfers (2e^−^˙*E*^0^ = 2.46 eV). Indeed, if one adds eqn (2) and (3), the result is: *OH + H_2_O → *OOH + 2H^+^ + 2e^−^. In this order of ideas, the second descriptor (*γ*_OOH/OH_) as defined in eqn (12), is a metric for the breaking of the scaling relation between the adsorption energies of *OOH and *OH from 3.20 to 2.46 eV:

12 γ_OOH/OH_ = (Δ*G*_OOH_ − Δ*G*_OH_ − 2*E*^0^)/2e^−^

This descriptor is worth considering, as the breaking of the *OOH–*OH scaling relation is usually prescribed to optimize OER electrocatalysts.^2,8,26^ The third descriptor is based on the concept of electrochemical symmetry. A fully symmetric catalyst, namely a thermodynamically ideal catalyst for the OER has: Δ*G*_1_ = Δ*G*_2_ = Δ*G*_3_ = Δ*G*_4_, which leads to *η*_OER_ = 0 and *γ*_OOH/OH_ = 0,^8,35^ according to eqn (11) and (12). For real catalysts, catalytic symmetry can be quantified in terms of the electrochemical-step symmetry index (ESSI):^36,37^13
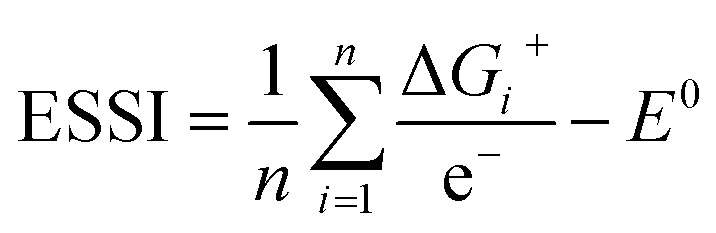
where Δ*G*_*i*_^+^ are the free energies of the electrochemical steps which are equal or larger than 1.23 eV along the OER pathway at 0 V *vs.* RHE, and in turn, *n* is the number of those steps. For example, let us consider the case of BaNiO_2_,^11^ which has Δ*G*_OH_ = 1.85 eV, Δ*G*_O_ = 3.46 eV and Δ*G*_OOH_ = 4.78 eV (see the ESI, Table S1†). With those we calculate that the free energies of the electrochemical steps are: Δ*G*_1_ = 1.85 eV, Δ*G*_2_ = 1.61 eV, Δ*G*_3_ = 1.32 eV, and Δ*G*_4_ = 0.14 eV. The PLS is step 1 and *η*_OER_ = 1.85 − 1.23 = 0.62 V. Steps 1, 2 and 3 are over 1.23 eV, so *n* = 3 and ESSI = (1.85 + 1.61 + 1.32)/3 − 1.23 = 0.36 V. In addition, *γ*_OOH/OH_ = (4.78 − 1.85 − 2.46)/2 = 0.24 V. We emphasise that for free energies falling close to 1.23 eV, some variations in the calculation setup (*e.g.*, the choice of a given exchange-correlation functional, energy cutoff, *k*-point sampling, ion-electron description) may induce integer changes in *n* and ESSI. A separate analysis is advisable for materials with such steps, instead of a general, statistical approach.

We note that *n* in eqn (13) can either be 1, 2 or 3 for real catalysts, while only the ideal catalyst has *n* = 4 and ESSI = 0. Importantly, in the next section we will show that *n* is a crucial parameter for the OER performance of electrocatalysts. In particular, catalysts with *n* = 3 will be statistically shown to most often display low values of *η*_OER_.

## Results and discussion

### How *n* influences the activity of catalysts

**Fig. 1:**
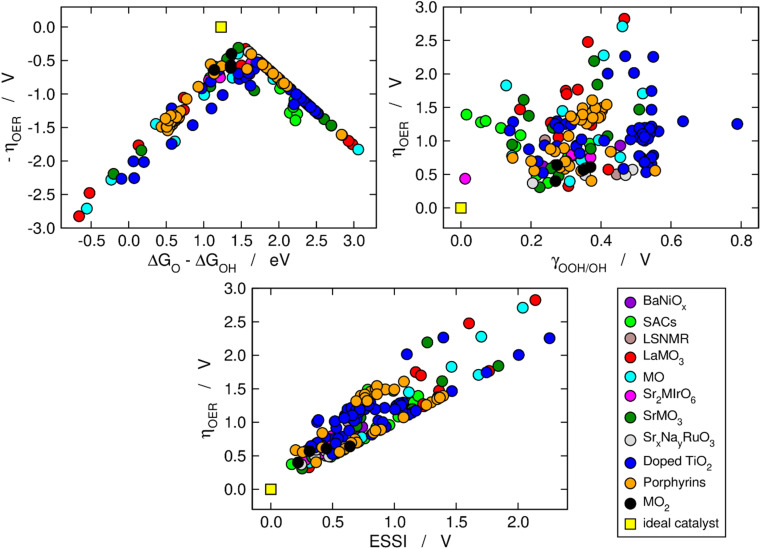
Computational description of OER activity trends on a variety of materials: BaNiO_*x*_,^11^ SACs,^5,6^ LSNMR,^14^ MO, LaMO_3_ and SrMO_3_,^7–9^ Sr_2_MIrO_6_,^13^ Sr_*x*_Na_*y*_RuO_3_,^12^ (doped) TiO_2_,^10^ metalloporphyrins,^15^ and MO_2_.^8^ Upper left corner: Sabatier-type activity plot showing −*η*_OER_ as a function of Δ*G*_O_ − Δ*G*_OH_. Upper right corner: *η*_OER_ plotted against the degree of breaking of the *OOH–*OH scaling relation (*γ*_OOH/OH_). Bottom: correlation between *η*_OER_ and the electrochemical-step symmetry index (ESSI).^36^ The ideal catalyst, for which *η*_OER_ = ESSI = *γ*_OOH/OH_ = 0, is shown in every panel for comparison.

We now use the three aforementioned descriptors to analyze the OER activity trends among the compounds in our database. In Fig. 1, the data are divided in families of materials, following the conventional depiction of oxygen evolution trends. The upper left panel is a Sabatier-type volcano plot, the apex of which is located at Δ*G*_O_ − Δ*G*_OH_ ≈ 1.60 eV and *η*_OER_ ≈ 0.37 V. For comparison, an ideal catalyst displays: Δ*G*_O_ − Δ*G*_OH_ ≈ 1.23 eV and *η*_OER_ ≈ 0, see the yellow square in Fig. 1. It has been argued that the volcano apex is found at these values in view of the transfer of more than two electrons during the OER catalytic cycle and the unfit energetic separation between *OOH and *OH close to 3.20 eV.^7,8,15,37,38^ Materials lying on the left side of the volcano (Δ*G*_O_ − Δ*G*_OH_ < 1.6 eV) bind the OER intermediates strongly, and step 3 (*O → *OOH) is the PLS. Conversely, materials on the right side of the volcano bind the OER intermediates weakly (Δ*G*_O_ − Δ*G*_OH_ > 1.6 eV), and the PLS is step 2 (*OH → *O).

The upper right panel of Fig. 1 shows no clear correlation between *η*_OER_ and *γ*_OOH/OH_, meaning that breaking the *OOH–*OH scaling relation does not guarantee a low OER overpotential.^36,37^ In fact, although such breaking is usually stipulated as the key to lowering OER overpotentials,^2,8,26^ the upper right panel of Fig. 1 calls for a rethinking, in line with previous works.^39^ Alternative quantitative strategies have been suggested, such as the delta-epsilon optimization^40^ and the partial breaking of the *OOH–*OH scaling relation.^41^

The bottom panel of Fig. 1 shows the proportional relationship between *η*_OER_ and ESSI. There are two features worth highlighting in this panel: first, it follows from eqn (11) and (13) that *η*_OER_ ≥ ESSI in all cases. The upper limit (ESSI = *η*_OER_) corresponds to catalysts with only one step above 1.23 eV (*n* = 1). Second, as ESSI approaches zero, which happens at low OER overpotentials, the dispersion among the data points decreases.^36^ Specifically, for ESSI ≈ 0.25 V the data points are in the range of 0.25–0.60 V, while for ESSI ≈ 1.50 V the data points are in the range of 1.50–2.30 V. This feature is encouraging, as it suggests that the predictive power of ESSI increases as the overpotentials tend to zero, that is, as materials become increasingly active.

**Fig. 2:**
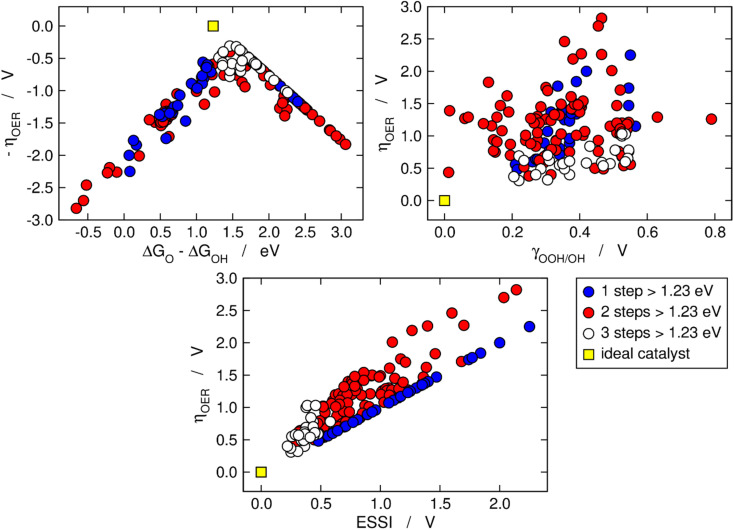
OER activity trends as in Fig. 1 but with the data classified depending on the number of electrochemical steps larger than 1.23 eV. Upper left corner: Sabatier-type activity plot for −*η*_OER_ as a function of Δ*G*_O_ − Δ*G*_OH_. Upper right corner: *η*_OER_ plotted against *γ*_OOH/OH_. Bottom: correlation between *η*_OER_ and ESSI. The ideal catalyst is shown in every panel for comparison.

Fig. 2 is analogous to Fig. 1, but the materials are classified by the number of electrochemical steps with free energies larger than 1.23 eV. On the upper left panel, materials with *n* = 3 appear to be concentrated around the top of the volcano, meaning that their OER overpotentials are low and relatively similar. This is not related to the breaking of the *OOH–*OH scaling relation, as the upper right panel shows that materials with *n* = 3 span a range of *γ*_OOH/OH_ between 0.2 and 0.6 V where numerous other materials with *n* = 1, 2 are also located that have large overpotentials. Conversely, the bottom panel of Fig. 2 clearly shows that materials with *n* = 3 generally display small ESSI values, suggesting that active OER materials tend to be electrochemically symmetric and *vice versa*, and that symmetry likely grows alongside *n*. We will come back to this point later in this manuscript.

**Fig. 3:**
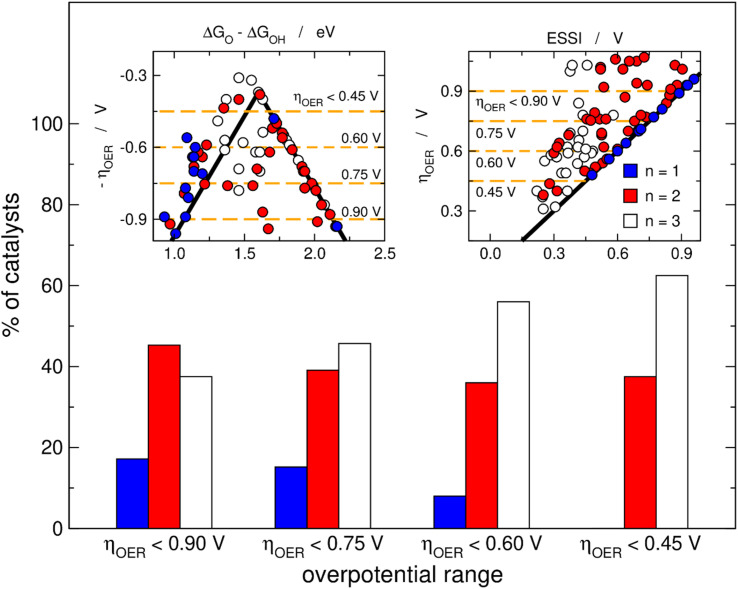
Proportion of OER catalysts with 1, 2 and 3 electrochemical steps over 1.23 eV for four different overpotential ranges. As the upper limit goes from 0.90, to 0.75, 0.60 and 0.45 V, the percentage of catalysts with *n* = 1 approaches zero, the percentage is relatively stable for catalysts with *n* = 2, while the percentage grows for catalysts with *n* = 3. Insets: volcano (left) and ESSI (right) plots with orange dashed lines indicating the upper limits of the overpotential ranges.

Fig. 3 helps in quantifying the general features of Fig. 2. Basically, we set four upper limits for the overpotential (0.90, 0.75, 0.60 and 0.45 V), and in each case we searched the number of materials with *n* = 1, 2, 3. The procedure is illustrated in the insets of Fig. 3 for the volcano and the ESSI plots, where the dashed lines correspond to the upper bounds of *η*_OER_. We observe that as the upper limit for the overpotential decreases, that is, as the catalysts become more active, the proportion of materials with *n* = 3 grows (from 38% for *η*_OER_ < 0.90 V to 63% for *η*_OER_ < 0.45 V), the proportion of materials with *n* = 2 decreases first and then remains relatively constant (from 45 to 38%), and the proportion of materials with *n* = 1 decreases (from 17 to 0%).

### Statistics of the reaction steps as a function of *n*

**Fig. 4:**
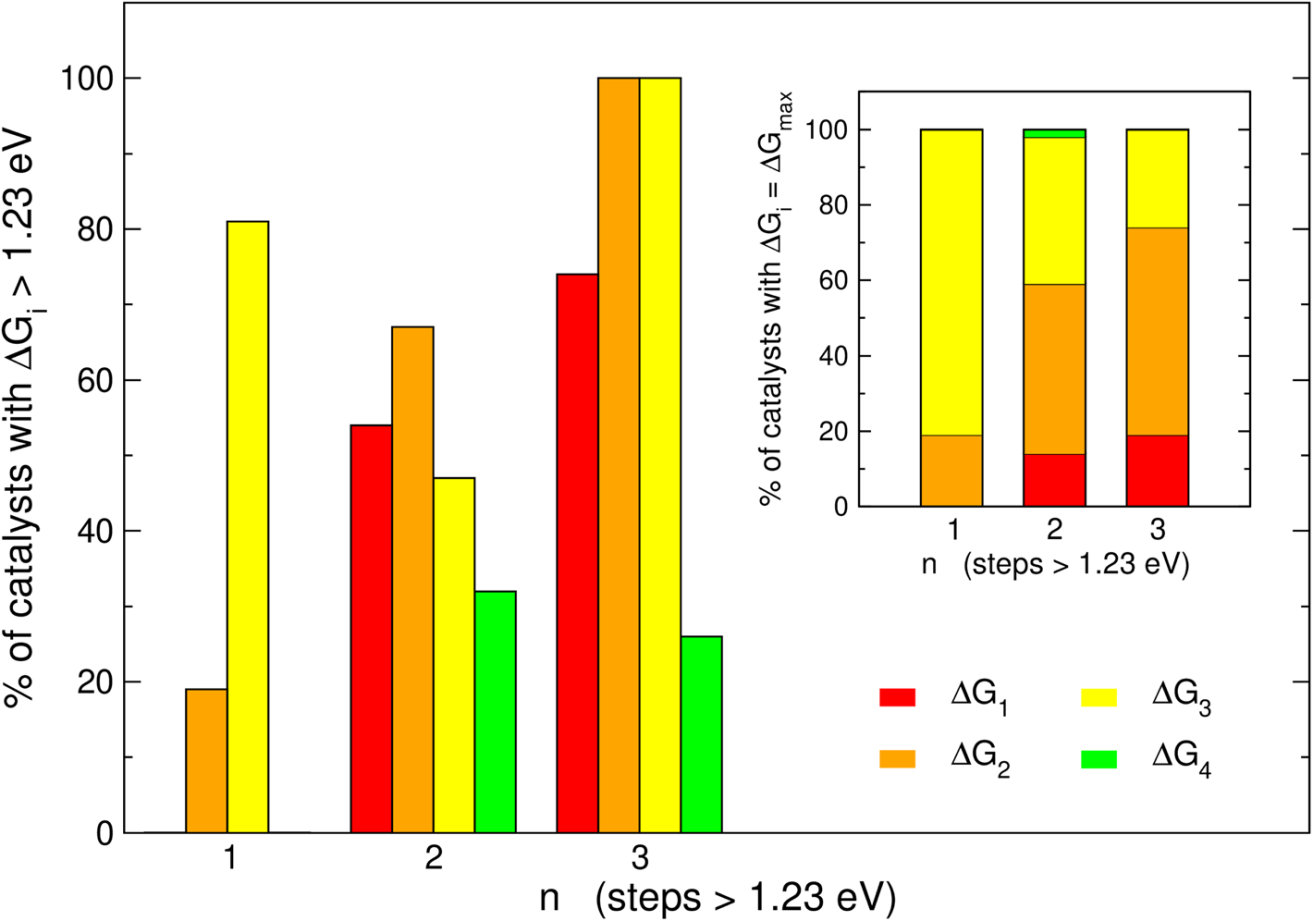
Proportion of the electrochemical steps 1–4 over 1.23 eV for catalysts with one, two and three electrochemical steps larger than 1.23 eV. For catalysts with *n* = 3, steps 2 and 3 are always larger than 1.23 eV, in 74% of the cases the other step over 1.23 eV is step 1, and in 26% of cases it is step 4. Inset: most common potential-limiting steps (PLSs). For catalysts with *n* = 1, the most common PLS is step 3; for catalysts with *n* = 2 and 3, the most common PLS is step 2 followed by step 3.

**Fig. 5:**
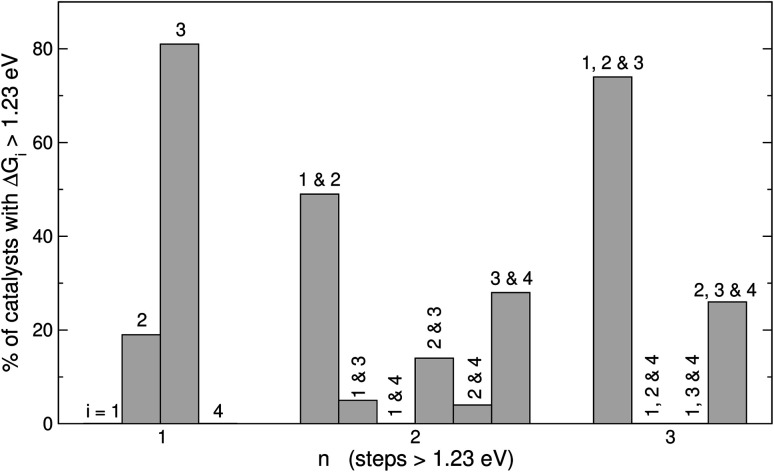
Combinations of OER steps larger than 1.23 eV. For catalysts with *n* = 1, step 3 is most often larger than 1.23 eV. For catalysts with *n* = 2, steps 1 and 2, and steps 3 and 4 are commonly over 1.23 eV. For catalysts with *n* = 3, steps 1, 2 and 3 are frequently altogether over 1.23 eV, whereas steps 2, 3 and 4 altogether are also frequently over 1.23 eV.

It is informative to know the most frequent reaction steps with energies over 1.23 eV when *n* = 1, 2, 3. We first analyze those steps separately in Fig. 4 and then altogether in Fig. 5. When *n* = 1, Fig. 4 shows step 3 (*O → *OOH) to be over 1.23 eV in 81% of the materials, in agreement with the upper left panel of Fig. 2 where most of the blue points are on the left of the volcano plot. Interestingly, when *n* = 1, no catalyst is limited by steps 1 or 4.

When *n* = 2 the most common step above 1.23 eV is step 2 (*OH → *O, 67%), followed by steps 1 and 3 (H_2_O → *OH and *O → *OOH, 54 and 47%, respectively). Finally, when *n* = 3 the most common steps above 1.23 eV are steps 1, 2 and 3 (74, 100 and 100%, respectively). Interestingly, this shows that when *n* = 3, steps 2 and 3 are always above 1.23 eV. Furthermore, the inset of Fig. 4 shows that the most frequent PLS is by far step 3 when *n* = 1 (81%), majorly steps 2 and 3 when *n* = 2 (45 and 39%), and step 2 when *n* = 3 (56%). Hence, steps 2 and 3 are usually potential-limiting and their proportions change in opposite directions as a function of *n*.

Fig. 5 displays the most frequent combinations of steps as a function of *n*. It is shown again that when *n* = 1, step 3 is the most likely step to be over 1.23 eV. For *n* = 2 the most frequent combination is that of steps 1 and 2 (49%), followed by the combination of steps 3 and 4 (28%). If *n* = 3, the combination of steps 1, 2, and 3 is found on most catalysts (74%), the combination of steps 2, 3, and 4 might be observed but to a lesser extent (26%), and other combinations are unlikely to happen. In perspective, Fig. 4 and 5 suggest that it is advisable to plan the optimization of catalysts according to *n*, and not simply aiming at breaking the *OOH–*OH scaling relation.

### Overall OER trends as a function of *n*

**Fig. 6:**
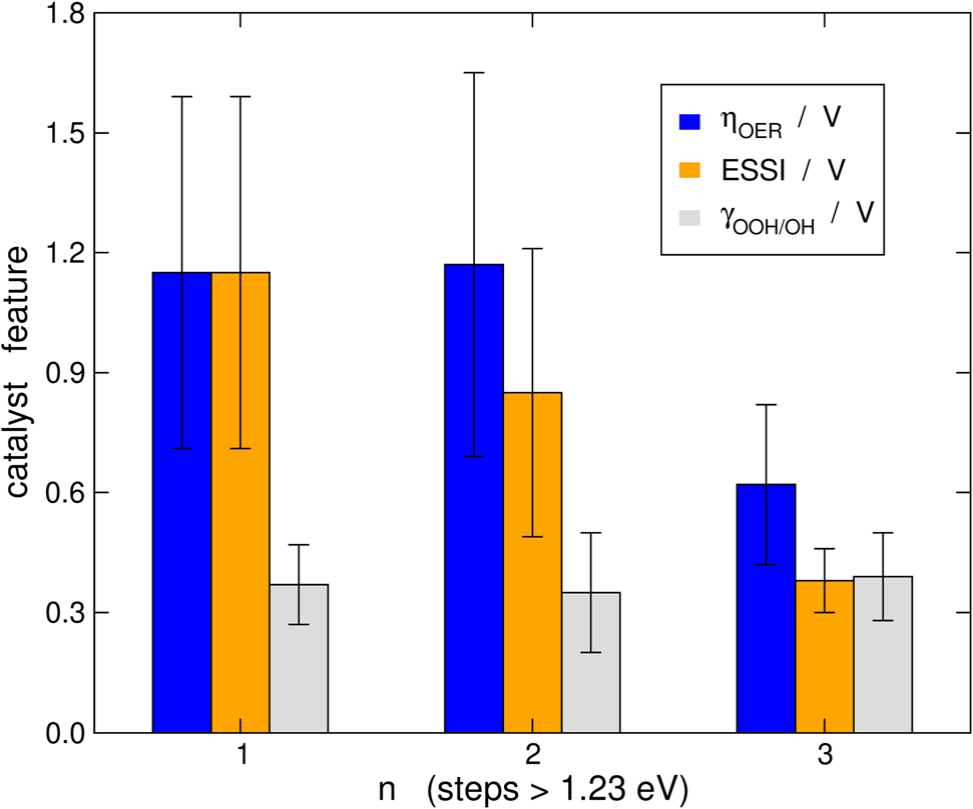
Average values of *η*_OER_, ESSI and *γ*_OOH/OH_ for catalysts with one, two and three electrochemical steps above 1.23 eV. The error bars correspond to the standard deviation of the data with respect to the means. Catalysts with *n* = 3 display lower overpotentials and ESSI values than the rest, and their error bars are narrower.

It is probably illustrative to visualize the overall trends in our data set at this point. To this end, in Fig. 6 we report the averages of *η*_OER_, *γ*_OOH/OH_, and ESSI for the cases in which *n* = 1, 2, 3. The error bars correspond to the standard deviations of the data. We reiterate that *γ*_OOH/OH_ may not be a good descriptor for the OER trends, as there are no appreciable changes in its average as a function of *n* and the error bars are of similar sizes. It is important to note in Fig. 6 that catalysts with *n* = 1, 2 span a broad range of overpotentials and ESSI values, hence the wide error bars. Admittedly, the entire groups of catalysts with *n* = 1, 2 are probably not well represented by their average. Nevertheless, for *n* = 3 the error bars are narrow and the average values of *η*_OER_ and ESSI are low. This supports our conclusion that catalysts with *n* = 3 are consistently more symmetric than the rest, exhibit lower overpotentials and their good OER performance does not stem from breaking the *OOH–*OH scaling relation. We note in passing that a catalyst with *n* = 1, 2 may reach *n* = 3 by means of delta-epsilon optimizations.^40^ Those provide in simple terms quantitative guidelines for the lowering of *η*_OER_ by weakening or strengthening the adsorption energies of *O, *OH and/or *OOH. Such guidelines can be implemented in experiments by means of tensile or compressive strain, ligand effects, electrolyte effects, ensemble effects, *etc.* (see ref. 40 and references therein).

### Connection to the free-energy span model

Recent works have highlighted the insights offered by the free-energy span model in electrocatalysis.^42–44^ The model is based on the largest positive free-energy difference between the species involved in a given catalytic pathway. Interestingly, the model can be used to anticipate the kinetic behavior of a catalyst on the basis of its reaction energies. We calculated the energy span of each catalyst in our database following the procedure in previous works^42,43^ (see Section S2†). We find that the most frequent span for catalysts with *n* = 1 is between *OOH and *O (Δ*G*_3_), which involves one electron transfer. In turn, the most frequent span for catalysts with *n* = 2 is between *O and the clean surface (Δ*G*_1_ + Δ*G*_2_), which involves two electron transfers. Finally, the most frequent span for catalysts with *n* = 3 is between *OOH and the clean surface (Δ*G*_1_ + Δ*G*_2_ + Δ*G*_3_), which involves three electron transfers. We note that the spans agree with the findings presented in Fig. 4, where we provided a statistical analysis of the relationship between *n* and the reaction free energies.

Furthermore, at a potential of 1.23 V *vs.* RHE (*η*_OER_ = 0) the average values of the span, denoted *G*_max_(*η*_OER_ = 0),^42,43^ are 1.15 ± 0.44, 1.68 ± 0.72, and 1.14 ± 0.25 eV for materials with *n* = 1, 2, 3, respectively. Because of the number of electrons transferred within the span, as the overpotential grows, the average *G*_max_(*η*_OER_) should decrease faster as a function of *n*. Indeed, when the applied potential is 1.60 V *vs.* RHE (*η*_OER_ = 0.37 V) the average values of *G*_max_(*η*_OER_ = 0.37 V) are 0.78 ± 0.44, 1.03 ± 0.67, and 0.27 ± 0.21 eV for materials with *n* = 1, 2, 3, respectively. In sum, more elaborate approaches such as the free-energy span model also show that materials with *n* = 3 are statistically more auspicious for the OER than materials with *n* = 1, 2.

## Conclusions

The general features of OER electrocatalysts that make them active are not overly clear in the state of the art, which limits our ability to perform well-informed searches for new materials. Experimentally, the main guideline is the addition of Ru or Ir (or Ni/Fe in alkaline media), and in computational terms the main recipes are to have Δ*G*_O_ − Δ*G*_OH_ ≈ 1.60 eV and supposedly breaking the *OOH–*OH scaling relation.

Here, a statistical analysis of nearly 160 DFT-optimized data points gathered from the literature led us to conclude that an active OER material typically has *n* = 3. In other words, three out of four OER electrochemical steps have free energies above 1.23 eV. Statistically, the three steps are steps 1 (H_2_O → *OH), 2 (*OH → *O), and 3 (*O → *OOH). The most likely potential-limiting step of an active OER material with *n* = 3 is step 2 (*OH → *O). Furthermore, materials with *n* = 3 are considerably more electrochemically symmetric than others, and their average ESSI and overpotential are descriptive of the overall class of materials, which is not the case for *n* = 1, 2. Finally, we found no connection between the breaking of the *OOH–*OH scaling relation and low overpotentials.

The implications of these findings are, first, that electrochemical symmetry is a suitable design criterion for OER electrode materials, and second, that the optimization of an OER catalyst with *n* = 1, 2 should aim for and is likely to entail an increase of *n*. Specific knowledge of the steps already above 1.23 eV and those close to that value provide a good starting point to devise effective, data-driven strategies for the enhancement of OER electrocatalysts.

## Data availability

The free energies of *O, *OH, *OOH and of steps 1, 2, 3, and 4 of the OER, together with the values of *η*_OER_, ESSI, *γ*_OOH/OH_ and the equations and results of the free-energy span model are provided in the ESI.†

## Author contributions

F. C. V. and F. I. conceived the idea behind this work. All authors contributed to the analysis of the data. E. R. and F. C. V. wrote the first draft of the manuscript and all authors edited its subsequent versions. F. I. and F. C. V. supervised the research work.

## Conflicts of interest

There are no conflicts to declare.

## Supplementary Material

SC-014-D2SC06832J-s001
