# Supplementary material for: A general but still unknown characteristic of active oxygen evolution electrocatalysts
Source: Chem Sci. 2023 Mar 7;14(13):3622–9. doi: 10.1039/d2sc06832j (PMC10056041; doi:10.1039/d2sc06832j)
Supplement: SC-014-D2SC06832J-s001 [file SC-014-D2SC06832J-s001.pdf]

Electronic Supplementary Material (ESI) for Chemical Science.
This journal is © The Royal Society of Chemistry 2023

Electronic Supplementary Information

A general but still unknown characteristic of active oxygen evolution electrocatalysts†

Eleonora Romeo,[a] Francesc Illas,[a,*] and Federico Calle-Vallejo[b,c,*]

[a] Department of Materials Science and Chemical Physics & Institute of Theoretical and Computational Chemistry (IQTCUB), University of Barcelona, C/ Martí i Franquès 1, 08028 Barcelona, Spain.

[b] Nano-Bio Spectroscopy Group and European Theoretical Spectroscopy Facility (ETSF), Department of Polymers and Advanced Materials: Physics, Chemistry and Technology, University of the Basque Country UPV/EHU, Av. Tolosa 72, 20018 San Sebastián, Spain.

[c] IKERBASQUE, Basque Foundation for Science, Plaza de Euskadi 5, 48009 Bilbao, Spain.

*Corresponding authors: FI (francesc.illas@ub.edu) and FCV (federico.calle@ehu.es)

Table of Contents

S1. Data collected from the literature 2

S2. Free-energy span model 8

References 9

S1. Data collected from the literature

We report in Tables S1 to S3 all the data collected from the literature, grouped by the number of electrochemical steps above 1.23 eV, i.e. $n = 1, 2, 3$. For each material, we specify the family it belongs to, as it is plotted in Figure 1 in the main text, with the relative color: $BaNiO_x$[1] in purple; single atom catalysts (SACs) [2,3] in light green; LSNMR[2] in brown; MO in light blue, $LaMO_3$ and $SrMO_3$[3–5] in red and dark green; Sr_2MIrO_6[6] in magenta; $Sr_xNa_yRuO_3$[7] in gray; undoped and doped TiO_2[8] in blue; porphyrins[9] in orange; and MO_2[4] in black.

The class of materials labelled $BaNiO_x$ includes two Ba-Ni-O phases, $BaNiO_2$ and $BaNiO_{2.78}$, with Ni atoms in square-planar and octahedral coordination, respectively.[1] SACs consist of 26 functionalized graphitic layers with MN_4 sites in which M = Cr, Mn, Fe, Co, Ni, Cu, Ru, Rh, Pd, Ag, Ir, Pt, Au. We note that there are two types of environments around those sites.[2,3] LSNMR is an acronym describing the double perovskite $La_{1.5}Sr_{0.5}NiMn_{0.5}Ru_{0.5}O_6$, a bifunctional OER/ORR catalyst belonging to the family of doped $AA'BB'O_6$ with different cations in the A and B sublattices, in this particular case La and Sr cations occupy the A and A' positions and Ni, Mn, and Ru cations occupy the B and B' positions.[2] MO are metal monoxides in the rock-salt geometry while $LaMO_3$ and $SrMO_3$ are perovskite oxides (AMO_3) with Sc, Ti, V, Cr, Mn, Fe, Co, Ni, Cu, Zn, Ru, Ga and Ge in the M position. [3–5] As part of the perovskite family, we also report Sr_2MIrO_6 which is a double perovskite,[6] and $Sr_xNa_yRuO_3$[7] which is a Na-doped ruthenium perovskite. The category "doped TiO_2" is composed of rutile TiO_2(110) with and without surface doping of V, Nb, Ta, Cr, Mo, W, Mn, Fe, Ru, Ir and Ni.[10] The porphyrin catalysts considered here have Cr, Mn, Fe, Co, Ni and Cu as metal center and -H, -F, -OH, $-NH_2$, $-CH_3$, and $-BH_2$ as ring ligands.[11] Lastly, we considered some dioxide materials, MO_2.[6]

Table S1. Free adsorption energy of adsorption (in eV) of *O, *OH and *OOH, *ESSI* (in V), a metric for the breaking of the *OOH-*OH scaling relation $\gamma_{OOH/OH}$ (in V), OER overpotential η_{OER} for materials with $n = 1$, I and II are 1.23 and 1.60 V vs RHE, respectively.

Material	family	ΔG_O	ΔG_{OH}	ΔG_{OOH}	$ESSI$	$\gamma_{OOH/OH}$	η_{OER}	$G_{max}(I)$	$G_{max}(II)$	span
V-TiO_2 (6cM)	u/d TiO_2	2.76	0.38	3.97	1.15	0.57	1.15	1.15	0.78	8
V-TiO_2 (6cM)	u/d TiO_2	3.40	1.02	4.56	1.15	0.54	1.15	1.15	0.78	8
V-TiO_2 (6cM)	u/d TiO_2	3.46	1.12	4.63	1.11	0.53	1.11	1.11	0.74	8
Nb-TiO_2 (6cM)	u/d TiO_2	2.69	0.29	3.80	1.17	0.53	1.17	1.17	0.80	8
Mo-TiO_2 (5cM)	u/d TiO_2	0.49	0.42	3.72	2.00	0.42	2.00	2.00	1.63	5
Mo-TiO_2 (5cM)	u/d TiO_2	1.46	0.61	4.16	1.47	0.55	1.47	1.47	1.10	5
Mo-TiO_2 (6cM)	u/d TiO_2	0.86	0.28	3.83	1.74	0.55	1.74	1.74	1.37	5
W-TiO_2 (5cM)	u/d TiO_2	0.42	0.34	3.90	2.25	0.55	2.25	2.25	1.88	5
Ru-TiO_2 (5cM)	u/d TiO_2	2.15	1.05	4.19	0.81	0.34	0.81	0.81	0.44	5
Mn-F	porphyrins	1.13	0.46	3.70	1.34	0.39	1.34	1.34	0.97	5
Mn-BH_2	porphyrins	1.41	0.75	3.94	1.30	0.37	1.30	1.30	0.93	5
Fe-H	porphyrins	1.19	0.63	3.77	1.35	0.34	1.35	1.35	0.98	5
Fe-F	porphyrins	1.37	0.68	3.85	1.25	0.36	1.25	1.25	0.88	5
Fe-CH_3	porphyrins	1.10	0.57	3.73	1.40	0.35	1.40	1.40	1.03	5
Fe-BH_2	porphyrins	1.51	0.88	4.09	1.35	0.38	1.35	0.56	0.19	5
Co-H	porphyrins	2.30	1.21	4.09	0.56	0.21	0.56	0.89	0.52	5
Co-F	porphyrins	1.97	1.04	4.09	0.89	0.30	0.89	1.07	0.70	5
Co-OH	porphyrins	1.65	0.88	3.95	1.07	0.31	1.07	0.70	0.33	5
Co-CH_3	porphyrins	2.14	1.00	4.07	0.70	0.31	0.70	1.37	1.00	5
Co-NH_2	porphyrins	1.26	0.77	3.86	1.37	0.32	1.37	1.84	1.47	5
$SrCrO_3$	$SrMO_3$	0.92	0.75	3.99	1.84	0.39	1.84	0.89	0.52	5
$SrMnO_3$	$SrMO_3$	2.29	1.21	4.41	0.89	0.37	0.89	0.60	0.23	5
$SrRuO_3$	$SrMO_3$	2.26	1.11	4.09	0.60	0.26	0.60	1.77	1.40	2
$LaCrO_3$	$LaMO_3$	0.69	0.56	3.69	1.77	0.34	1.77	1.23	0.86	5
$LaMnO_3$	$LaMO_3$	1.39	0.65	3.85	1.23	0.37	1.23	0.64	0.27	5
$LaRuO_3$	$LaMO_3$	1.87	0.74	3.74	0.64	0.27	0.64	0.71	0.34	5
CoO	MO	1.98	0.78	3.92	0.71	0.34	0.71	0.96	0.59	5
Mn-FGM	SACs	1.89	0.88	4.08	0.96	0.37	0.96	0.77	0.40	5
Fe-FGM	SACs	2.11	1.03	4.11	0.77	0.31	0.77	0.48	0.11	8
Co-FGM	SACs	2.93	1.22	4.11	0.48	0.22	0.48	0.93	0.56	8
LSNMR@Mn	LSNMR	3.12	0.96	4.08	0.93	0.33	0.93	0.64	0.27	5
PtO_2	rutiles	2.35	1.21	4.22	0.64	0.28	0.64	1.35	0.98	5

Table S2. Free adsorption energy of adsorption (in eV) of *O, *OH and *OOH, *ESSI* (in V), a metric for the breaking of the *OOH-*OH scaling relation $\gamma_{OOH/OH}$ (in V), OER overpotential η_{OER} for materials with $n = 2$, I and II are 1.23 and 1.60 V vs RHE, respectively.

material	family	ΔG_O	ΔG_{OH}	ΔG_{OOH}	$ESSI$	$\gamma_{OOH/OH}$	η_{OER}	$G_{max}(I)$	$G_{max}(II)$	span
TiO_2	u/d TiO_2	4.60	2.07	5.08	1.07	0.28	1.30	2.14	1.40	9
V-TiO_2 (5cM)	u/d TiO_2	2.35	1.38	4.50	0.54	0.33	0.92	0.92	0.55	5
V-TiO_2 (5cM)	u/d TiO_2	3.17	1.18	4.67	0.52	0.52	0.76	1.03	0.39	6
Nb-TiO_2 (5cM)	u/d TiO_2	2.14	0.23	3.74	0.53	0.53	0.68	1.05	0.31	6
Nb-TiO_2 (6cM)	u/d TiO_2	2.38	0.05	3.59	0.60	0.54	1.10	1.18	0.73	3
Ta-TiO_2 (6cM)	u/d TiO_2	2.17	-0.35	3.37	0.81	0.63	1.29	1.58	0.92	3
Ta-TiO_2 (6cM)	u/d TiO_2	2.55	0.13	3.64	0.62	0.53	1.20	1.20	0.83	8
Cr-TiO_2 (5cM)	u/d TiO_2	2.43	1.85	4.87	0.92	0.28	1.21	1.21	0.84	5
Cr-TiO_2 (6cM)	u/d TiO_2	2.91	1.23	4.76	0.54	0.54	0.62	1.07	0.33	6
Cr-TiO_2 (6cM)	u/d TiO_2	4.14	1.72	5.23	0.84	0.53	1.19	1.68	0.94	9
Cr-TiO_2 (6cM)	u/d TiO_2	3.84	1.56	5.04	0.69	0.51	1.05	1.38	0.68	9
Mo-TiO_2 (6cM)	u/d TiO_2	2.18	0.62	4.17	0.55	0.55	0.76	1.09	0.39	6
Mo-TiO_2 (6cM)	u/d TiO_2	2.35	0.58	4.10	0.53	0.53	0.54	1.06	0.32	6
W-TiO_2 (5cM)	u/d TiO_2	-0.33	-0.23	3.16	1.40	0.47	2.26	2.79	2.05	2
W-TiO_2 (6cM)	u/d TiO_2	0.26	0.05	3.50	1.10	0.50	2.01	2.20	1.64	2
W-TiO_2 (6cM)	u/d TiO_2	1.16	0.05	3.60	0.65	0.55	1.21	1.30	0.84	2
W-TiO_2 (6cM)	u/d TiO_2	1.46	0.21	3.71	0.52	0.52	1.02	1.04	0.65	6
Mn-TiO_2 (5cM)	u/d TiO_2	4.22	2.06	5.07	0.88	0.28	0.93	1.76	1.02	9
Mn-TiO_2 (6cM)	u/d TiO_2	4.20	1.94	5.09	0.87	0.35	1.03	1.74	1.00	9
Mn-TiO_2 (6cM)	u/d TiO_2	4.50	2.01	5.07	1.02	0.30	1.26	2.04	1.30	9
Mn-TiO_2 (6cM)	u/d TiO_2	4.48	2.01	5.10	1.01	0.32	1.24	2.02	1.28	9
Fe-TiO_2 (5cM)	u/d TiO_2	3.73	2.10	4.95	0.64	0.20	0.87	1.27	0.53	9
Fe-TiO_2 (5cM)	u/d TiO_2	4.31	2.16	5.10	0.93	0.24	0.93	1.85	1.11	9
Fe-TiO_2 (6cM)	u/d TiO_2	3.98	1.55	5.06	0.76	0.53	1.20	1.52	0.83	9
Fe-TiO_2 (5cM)	u/d TiO_2	3.88	1.50	4.94	0.71	0.49	1.15	1.42	0.78	9
Ru-TiO_2 (6cM)	u/d TiO_2	2.98	0.49	4.53	0.79	0.79	1.26	1.58	0.89	6
Ru-TiO_2 (6cM)	u/d TiO_2	3.83	1.42	4.96	0.69	0.54	1.18	1.37	0.81	9
Ru-TiO_2 (6cM)	u/d TiO_2	3.90	1.47	4.95	0.72	0.51	1.20	1.44	0.83	9
Ir-TiO_2 (5cM)	u/d TiO_2	1.81	0.61	3.68	0.33	0.31	0.64	0.65	0.27	2
Ir-TiO_2 (5cM)	u/d TiO_2	3.45	1.75	4.68	0.50	0.24	0.52	0.99	0.25	7
Ir-TiO_2 (6cM)	u/d TiO_2	2.72	1.13	4.64	0.53	0.53	0.69	1.05	0.32	6
Ir-TiO_2 (6cM)	u/d TiO_2	3.78	1.44	4.94	0.66	0.52	1.11	1.32	0.74	9
Ir-TiO_2 (6cM)	u/d TiO_2	3.64	1.35	4.86	0.59	0.53	1.06	1.18	0.69	9
Ni-TiO_2 (5cM)	u/d TiO_2	4.55	2.03	4.80	1.05	0.16	1.29	2.09	1.35	9
Ni-TiO_2 (5cM)	u/d TiO_2	4.59	2.39	5.12	1.06	0.14	1.16	2.13	1.39	9
Ni-TiO_2 (6cM)	u/d TiO_2	4.27	2.03	5.31	0.91	0.41	1.01	1.81	1.07	9
Ni-TiO_2 (6cM)	u/d TiO_2	4.57	2.08	5.08	1.06	0.27	1.26	2.11	1.37	9
Ni-TiO_2 (6cM)	u/d TiO_2	4.47	2.04	5.06	1.01	0.28	1.20	2.01	1.27	9
Cr-H	porphyrins	0.85	0.26	3.53	0.81	0.41	1.45	1.61	1.08	2

Cr-F	porphyrins	1.00	0.38	3.63	0.73	0.40	1.40	1.46	1.03	2
Cr-OH	porphyrins	0.76	0.22	3.49	0.85	0.41	1.50	1.70	1.13	2
Cr-CH_3	porphyrins	0.61	0.18	3.33	0.93	0.35	1.49	1.85	1.12	2
Cr-BH_2	porphyrins	0.94	0.36	3.58	0.76	0.38	1.41	1.52	1.04	2
Cr-NH_2	porphyrins	0.60	0.10	3.33	0.93	0.39	1.50	1.86	1.13	2
Mn-H	porphyrins	0.93	0.27	3.53	0.77	0.40	1.37	1.53	1.00	2
Mn-OH	porphyrins	0.74	0.23	3.51	0.86	0.41	1.54	1.72	1.17	2
Mn-CH_3	porphyrins	0.74	0.18	3.39	0.86	0.38	1.42	1.72	1.05	2
Mn-NH_2	porphyrins	0.46	-0.05	3.18	1.00	0.39	1.49	2.00	1.26	2
Fe-OH	porphyrins	1.07	0.48	3.62	0.70	0.34	1.32	1.39	0.95	2
Fe-NH_2	porphyrins	0.89	0.34	3.45	0.79	0.33	1.33	1.57	0.96	2
Co-BH_2	porphyrins	2.47	1.24	4.29	0.30	0.30	0.59	0.60	0.22	7
Ni-H	porphyrins	3.66	1.82	4.85	0.60	0.29	0.61	1.20	0.46	9
Ni-CH_3	porphyrins	3.82	1.84	4.60	0.68	0.15	0.75	1.36	0.62	9
Ni-BH_2	porphyrins	3.85	1.93	4.78	0.70	0.20	0.70	1.39	0.65	9
Cu-H	porphyrins	4.12	2.07	5.07	0.83	0.27	0.84	1.66	0.92	9
Cu-F	porphyrins	4.15	2.04	5.01	0.85	0.26	0.88	1.69	0.95	9
Cu-CH_3	porphyrins	3.76	1.83	4.89	0.65	0.30	0.70	1.30	0.56	9
Cu-BH_2	porphyrins	4.61	1.78	4.99	1.08	0.37	1.61	2.15	1.41	9
Cu-NH_2	porphyrins	2.69	0.92	4.48	0.55	0.55	0.56	1.10	0.36	6
$SrScO_3$	$SrMO_3$	5.23	2.38	5.21	1.39	0.19	1.62	2.77	2.03	9
$SrTiO_3$	$SrMO_3$	3.91	1.61	4.90	0.73	0.42	1.07	1.45	0.71	9
$SrVO_3$	$SrMO_3$	-0.07	0.13	3.35	1.27	0.38	2.19	2.53	1.82	2
$SrNiO_3$	$SrMO_3$	3.84	2.17	4.92	0.69	0.15	0.94	1.38	0.64	9
$SrCuO_3$	$SrMO_3$	4.75	2.32	5.31	1.15	0.27	1.20	2.29	1.55	9
$SrZnO_3$	$SrMO_3$	5.16	2.56	5.40	1.35	0.19	1.37	2.70	1.96	9
$SrGeO_3$	$SrMO_3$	4.44	1.74	5.22	0.99	0.51	1.47	1.98	1.24	9
$LaScO_3$	$LaMO_3$	4.80	1.82	4.88	1.17	0.30	1.75	2.34	1.60	9
$LaTiO_3$	$LaMO_3$	-1.82	-1.16	2.23	2.14	0.47	2.82	4.28	3.54	2
$LaVO_3$	$LaMO_3$	-0.74	-0.22	2.95	1.60	0.36	2.46	3.20	2.46	2
$LaCoO_3$	$LaMO_3$	2.22	1.49	4.50	0.66	0.28	1.05	1.05	0.68	5
$LaCuO_3$	$LaMO_3$	4.92	2.42	5.39	1.23	0.26	1.27	2.46	1.72	9
$LaZnO_3$	$LaMO_3$	5.18	2.48	5.27	1.36	0.17	1.47	2.72	1.98	9
$LaGaO_3$	$LaMO_3$	4.88	1.95	5.02	1.21	0.31	1.70	2.42	1.68	9
CaO	MO	5.38	2.32	5.04	1.46	0.13	1.83	2.92	2.18	9
ScO	MO	-0.90	-1.52	1.98	1.68	0.52	1.71	3.36	2.62	2
TiO	MO	-1.61	-1.05	2.32	2.04	0.46	2.70	4.07	3.33	2
VO	MO	-0.94	-0.71	2.56	1.70	0.41	2.27	3.40	2.66	2
CrO	MO	0.22	-0.13	2.90	1.12	0.29	1.45	2.24	1.50	2
MnO	MO	1.42	0.42	3.66	0.52	0.39	1.01	1.04	0.64	2
FeO	MO	1.76	0.38	3.75	0.46	0.46	0.76	0.91	0.39	6
NiO	MO	2.49	1.03	4.12	0.32	0.32	0.40	0.63	0.03	6
CuO	MO	3.93	2.00	4.75	0.74	0.15	0.77	1.47	0.73	9
Cr-FGM	SACs	0.89	0.35	3.60	0.79	0.40	1.48	1.57	1.11	2

Ni-FGM	SACs	4.16	2.14	4.91	0.85	0.16	0.91	1.70	0.96	9
Cu-FGM	SACs	4.52	2.31	5.11	1.03	0.17	1.08	2.06	1.32	9
Ru-FGM	SACs	1.72	0.58	3.63	0.37	0.30	0.68	0.74	0.31	2
Rh-FGM	SACs	2.84	1.23	4.19	0.25	0.25	0.38	0.50	0.01	6
Pd-FGM	SACs	4.65	2.50	5.08	1.10	0.06	1.27	2.19	1.45	9
Ag-FGM	SACs	4.77	2.52	5.12	1.16	0.07	1.29	2.31	1.57	9
Ir-FGM	SACs	2.41	1.24	4.26	0.32	0.28	0.62	0.62	0.25	5
Pt-FGM	SACs	4.61	2.42	5.11	1.08	0.12	1.19	2.15	1.41	9
Au-FGM	SACs	4.84	2.62	5.11	1.19	0.02	1.39	2.38	1.64	9
$BaNiO_3$	$BaNiO_x$	3.88	1.87	4.99	0.71	0.33	0.78	1.42	0.68	9
$BaNiO_{3-d2}$	$BaNiO_x$	3.94	1.78	5.15	0.74	0.46	0.93	1.48	0.74	9
$Sr_5Na_2Ru_8O_{24}$	$Sr_xNa_yRuO_3$	2.66	0.94	4.34	0.47	0.47	0.49	0.94	0.20	6
LSNMR@Ni	LSNMR	3.71	1.47	4.41	0.63	0.24	1.01	1.25	0.64	9
LSNMR@Ru	LSNMR	2.27	0.54	3.89	0.45	0.45	0.50	0.89	0.15	6
Sr_2NiIrO_6	Sr_2MIrO_6	2.12	0.77	3.25	0.28	0.01	0.44	0.46	0.07	3
Sr_2ScIrO_6	Sr_2MIrO_6	1.48	0.41	3.50	0.49	0.32	0.79	0.98	0.42	2
Sr_2FeIrO_6	Sr_2MIrO_6	1.51	0.30	3.50	0.47	0.37	0.75	0.95	0.38	2

Table S3. Free adsorption energy of adsorption (in eV) of *O, *OH and *OOH, *ESSI* (in V), a metric for the breaking of the *OOH-*OH scaling relation $\gamma_{OOH/OH}$ (in V), OER overpotential η_{OER} for materials with $n = 3$, I and II are 1.23 and 1.60 V vs RHE, respectively.

material	family	ΔG_O	ΔG_{OH}	ΔG_{OOH}	*ESSI*	$\gamma_{OOH/OH}$	η_{OER}	$G_{max}(I)$	$G_{max}(II)$	span
Nb-TiO_2 (5cM)	u/d TiO_2	1.38	-0.08	3.39	0.44	0.51	0.78	1.31	0.41	3
Nb-TiO_2 (6cM)	u/d TiO_2	2.33	0.10	3.61	0.38	0.53	1.00	1.13	0.63	3
Ta-TiO_2 (5cM)	u/d TiO_2	1.50	-0.51	3.04	0.58	0.55	0.78	1.74	0.63	3
Ta-TiO_2 (5cM)	u/d TiO_2	1.86	-0.02	3.52	0.42	0.54	0.65	1.25	0.34	3
Ta-TiO_2 (6cM)	u/d TiO_2	2.33	0.07	3.60	0.39	0.54	1.03	1.16	0.66	3
Cr-TiO_2 (5cM)	u/d TiO_2	3.44	1.61	5.14	0.48	0.54	0.60	1.45	0.34	7
Mn-TiO_2 (5cM)	u/d TiO_2	3.38	1.92	4.82	0.38	0.22	0.69	1.13	0.32	7
Fe-TiO_2 (6cM)	u/d TiO_2	3.81	1.55	5.06	0.46	0.53	1.03	1.37	0.66	7
Ru-TiO_2 (5cM)	u/d TiO_2	3.55	1.73	5.12	0.48	0.47	0.59	1.43	0.35	7
Ni-F	porphyrins	3.43	1.85	4.93	0.41	0.31	0.62	1.24	0.25	7
Ni-OH	porphyrins	3.21	1.58	4.78	0.36	0.37	0.40	1.09	0.03	7
Ni-NH_2	porphyrins	3.10	1.32	4.46	0.26	0.34	0.55	0.77	0.18	7
Cu-OH	porphyrins	3.69	1.62	4.94	0.42	0.43	0.84	1.25	0.49	7
$SrFeO_3$	$SrMO_3$	2.98	1.67	4.70	0.34	0.29	0.49	1.01	0.12	7
$SrCoO_3$	$SrMO_3$	3.00	1.54	4.44	0.25	0.22	0.31	0.75	-0.06	7
$LaFeO_3$	$LaMO_3$	2.74	1.25	4.55	0.29	0.42	0.58	0.86	0.21	7
$LaNiO_3$	$LaMO_3$	3.09	1.54	4.61	0.31	0.31	0.32	0.92	-0.05	7
$BaNiO_{3-d1}$	$BaNiO_x$	3.54	1.93	5.00	0.44	0.31	0.70	1.31	0.34	7
$BaNiO_2$	$BaNiO_x$	3.46	1.85	4.78	0.36	0.24	0.62	1.09	0.26	7
$Sr_{7/8}Na_{1/8}RuO_3$	$Sr_xNa_yRuO_3$	3.16	1.56	4.43	0.25	0.21	0.37	0.74	0.00	7
$Sr_{6/8}Na_{2/8}RuO_3$	$Sr_xNa_yRuO_3$	3.31	1.59	4.76	0.36	0.36	0.49	1.07	0.12	7
$Sr_7Ru_8O_{24}$	$Sr_xNa_yRuO_3$	3.38	1.58	5.02	0.44	0.49	0.57	1.33	0.24	7
$Sr_6Na_1Ru_8O_{24}$	$Sr_xNa_yRuO_3$	3.44	1.62	4.79	0.37	0.36	0.59	1.10	0.24	7
IrO_2	rutiles	1.65	0.29	3.45	0.31	0.35	0.57	0.94	0.20	3
RuO_2	rutiles	2.72	1.35	4.35	0.22	0.27	0.40	0.66	0.03	7
MnO_2	rutiles	3.20	1.84	5.04	0.45	0.37	0.61	1.35	0.24	7
Sr_2CoIrO_6	Sr_2MIrO_6	1.63	0.00	3.40	0.41	0.47	0.54	1.23	0.20	3

S2. Free-energy span model

The potential-dependent free-energy spans are calculated on the basis of the adsorption energies of *O, *OH and *OOH as follows:[10]

$$span_1(U) = \Delta G_{O_2}(U) - \Delta G_{OOH}(U) \quad \text{(S1)}$$

$$span_2(U) = \Delta G_{O_2}(U) - \Delta G_{O}(U) \quad \text{(S2)}$$

$$span_3(U) = \Delta G_{O_2}(U) - \Delta G_{OH}(U) \quad \text{(S3)}$$

$$span_4(U) = \Delta G_{O_2}(U) - \Delta G_{H_2O}(U) \quad \text{(S4)}$$

$$span_5(U) = \Delta G_{OOH}(U) - \Delta G_{O}(U) \quad \text{(S5)}$$

$$span_6 = \Delta G_{OOH}(U) - \Delta G_{OH}(U) \quad \text{(S6)}$$

$$span_7(U) = \Delta G_{OOH}(U) - \Delta G_{H_2O}(U) \quad \text{(S7)}$$

$$span_8(U) = \Delta G_{O}(U) - \Delta G_{OH}(U) \quad \text{(S8)}$$

$$span_9(U) = \Delta G_{O}(U) - \Delta G_{H_2O}(U) \quad \text{(S9)}$$

$$span_{10}(U) = \Delta G_{OH}(U) - \Delta G_{H_2O}(U) \quad \text{(S10)}$$

where $\Delta G_{H_2O} = 0$ at all potentials and $\Delta G_{O_2} = 4.92\ eV$ at 0 V vs RHE. The effect of the potential on the adsorption energies is evaluated by means of the computational hydrogen electrode.[11] The descriptor $G_{max}(\eta)$ is calculated as the largest positive span in Equations S1-S10:

$$G_{max}(\eta) = \max\,(span_i(U)) \quad \text{(S11)}$$

References

1 M. Retuerto, F. Calle-Vallejo, L. Pascual, P. Ferrer, Á. García, J. Torrero, D. Gianolio, J. L. G. Fierro, M. A. Peña, J. A. Alonso and S. Rojas, Role of lattice oxygen content and Ni geometry in the oxygen evolution activity of the Ba-Ni-O system, *J. Power Sources*, 2018, **404**, 56–63.

2 M. Retuerto, F. Calle-Vallejo, L. Pascual, G. Lumbeeck, M. T. Fernandez-Diaz, M. Croft, J. Gopalakrishnan, M. A. Peña, J. Hadermann, M. Greenblatt and S. Rojas, La1.5Sr0.5NiMn0.5Ru0.5O6 Double Perovskite with Enhanced ORR/OER Bifunctional Catalytic Activity, *ACS Appl. Mater. Interfaces*, 2019, **11**, 21454–21464.

3 F. Calle-Vallejo, N. G. Inoglu, H. Y. Su, J. I. Martínez, I. C. Man, M. T. M. Koper, J. R. Kitchin and J. Rossmeisl, Number of outer electrons as descriptor for adsorption processes on transition metals and their oxides, *Chem. Sci.*, 2013, **4**, 1245–1249.

4 I. C. Man, H. Y. Su, F. Calle-Vallejo, H. A. Hansen, J. I. Martínez, N. G. Inoglu, J. Kitchin, T. F. Jaramillo, J. K. Nørskov and J. Rossmeisl, Universality in Oxygen Evolution Electrocatalysis on Oxide Surfaces, *ChemCatChem*, 2011, **3**, 1159–1165.

5 F. Calle-Vallejo, O. A. Díaz-Morales, M. J. Kolb and M. T. M. Koper, Why is bulk thermochemistry a good descriptor for the electrocatalytic activity of transition metal oxides?, *ACS Catal*, 2015, **5**, 869–873.

6 M. Retuerto, L. Pascual, O. Piqué, P. Kayser, M. A. Salam, M. Mokhtar, J. A. Alonso, M. Peña, F. Calle-Vallejo and S. Rojas, How oxidation state and lattice distortion influence the oxygen evolution activity in acid of iridium double perovskites, *J. Mater. Chem. A*, 2021, **9**, 2980–2990.

7 M. Retuerto, L. Pascual, F. Calle-Vallejo, P. Ferrer, D. Gianolio, A. G. Pereira, Á. García, J. Torrero, M. T. Fernández-Díaz, P. Bencok, M. A. Peña, J. L. G. Fierro and S. Rojas, Na-doped ruthenium perovskite electrocatalysts with improved oxygen evolution activity and durability in acidic media, *Nat. Commun. 2019 10:1*, 2019, **10**, 1–9.

8 M. García-Mota, A. Vojvodic, H. Metiu, I. C. Man, H. Y. Su, J. Rossmeisl and J. K. Nørskov, Tailoring the Activity for Oxygen Evolution Electrocatalysis on Rutile TiO2(110) by Transition-Metal Substitution, *ChemCatChem*, 2011, **3**, 1607–1611.

9 F. Calle-Vallejo, A. Krabbe and J. M. García-Lastra, How covalence breaks adsorption-energy scaling relations and solvation restores them, *Chem. Sci.*, 2016, **8**, 124–130.

10 S. Razzaq and K. S. Exner, Materials Screening by the Descriptor Gmax(η): The Free-Energy Span Model in Electrocatalysis, *ACS Catal.*, 2023, 1740–1758.

11 J. K. Nørskov, J. Rossmeisl, A. Logadottir, L. Lindqvist, J. R. Kitchin, T. Bligaard and H. Jónsson, Origin of the Overpotential for Oxygen Reduction at a Fuel-Cell Cathode, *J. Phys. Chem. B*, 2004, **108**, 17886–17892.
